# *OXTR* polymorphisms associated with severity and treatment responses of schizophrenia

**DOI:** 10.1038/s41537-023-00413-5

**Published:** 2024-01-06

**Authors:** Xue Lv, Yue-Sen Hou, Zhao-Hui Zhang, Wei-Hua Yue

**Affiliations:** 1 The First Affiliated Hospital of Xinxiang Medical College, 453100 Xinxiang, Henan, China; 2 Henan Key Laboratory of Neurorestoratology, 453199 Xinxiang, Henan China; 3 Henan Engineering Research Center of Physical Diagnostics and Treatment Technology for the Mental and Neurological Diseases, 453005 Xinxiang, Henan China; 4 Peking University Sixth Hospital, Peking University Institute of Mental Health, 100191 Beijing, China

**Keywords:** Genetics of the nervous system, Schizophrenia

## Abstract

The mechanisms generating specific symptoms of schizophrenia remain unclear and genetic research makes it possible to explore these issues at a fundamental level. Taking into account the associations between the oxytocin system and social functions, which are apparently impaired in schizophrenia patients, we hypothesized that the oxytocin receptor gene (*OXTR*) might be associated with schizophrenia symptoms in both severity and responses to antipsychotics and did this exploratory positional study. A total of 2363 patients with schizophrenia (1181 males and 1182 females) included in our study were randomly allocated to seven antipsychotic treatment groups and received antipsychotic monotherapy for 6 weeks. Their blood DNA was genotyped for *OXTR* polymorphisms. Their symptom severity was assessed by Positive and Negative Syndrome Scale (PANSS), and the scores were transformed into seven factors (positive, disorganized, negative symptoms apathy/avolition, negative symptoms deficit of expression, hostility, anxiety and depression). Percentage changes in PANSS scores from baseline to week 6 were calculated to quantify antipsychotic responses. We found that *OXTR* polymorphisms were nominally associated with the severity of overall symptoms (rs237899, β = 1.669, *p* = 0.019), hostility symptoms (rs237899, β = 0.427, *p* = 0.044) and anxiety symptoms (rs13316193, β = −0.197, *p* = 0.038). As for treatment responses, *OXTR* polymorphisms were nominally associated with the improvement in negative symptoms apathy/avolition (rs2268490, β = 2.235, *p* = 0.0499). No association between severity or response to treatment and *OXTR* polymorphisms was found with statistical correction for multiplicity. Overall, our results highlighted the possibility of nominally significant associations of the *OXTR* gene with the severity and improvement in schizophrenia symptoms. Given the exploratory nature of this study, these associations are indicative of the role of the *OXTR* gene in the pathology of schizophrenia and may contribute to further elucidate the mechanism of specific symptoms of schizophrenia and to exploit antipsychotics more effective to specific symptoms.

## Introduction

Schizophrenia, one of the most serious of all psychiatric illnesses, has a lifetime prevalence of around 1% worldwide. Patients with schizophrenia commonly present with positive symptoms (e.g., delusions, hallucinations, and formal thought disorder), negative symptoms (e.g., lack of volition, reduced speech output, and flattening of affect) and cognition impairment, particularly in executive function, memory, and sustained attention^1^.

The causes of schizophrenia have been studied for many years. Genome-wide association studies identified that schizophrenia is a polygenic disorder, making possible further elucidation of this disease at the genetic level^2,3^. Moreover, individual responses to antipsychotics, the primary treatment for schizophrenia, vary widely^4^. Genome-wide association studies also provide evidence for the involvement of genetic factors in between-patient variations^5^. In spite of this, to date, how risk loci beget specific symptoms of schizophrenia and variable responses to antipsychotics remains unclear.

Oxytocin receptor (OXTR), the key member of the oxytocin-signaling pathway, has been identified to get involved in human social functions, the impairments of which are core features of most psychiatric illnesses like schizophrenia^6,7^. Genetic research has demonstrated that allelic variants of *OXTR* give rise to tendencies for individuals to behave differently in social relationships. For example, among schizophrenia patients, compared to *OXTR* rs53576 GG homozygous subjects, *OXTR* rs53576 A carriers had more empathic concern^8^; the severity of schizophrenia symptoms was associated with variants in the *OXTR* (rs237885, rs237887)^9^. Such findings support that *OXTR* polymorphisms could play a major role in the neurobiology of generating different symptoms of schizophrenia. Besides, *OXTR* polymorphisms have been found to be associated with improvement in symptoms of schizophrenia following antipsychotic agents. In patients treated with clozapine, variants in the *OXTR* were nominally associated with the improvement in the positive symptoms (rs11706648, rs4686301, rs237899)^9^. However, previous studies on the association between *OXTR* polymorphisms and schizophrenia usually focused on general symptoms but specific symptoms, studied limited antipsychotics, and had small sample sizes.

To further investigate how *OXTR* polymorphisms impact on severity of multidimensional symptoms of schizophrenia and responses to antipsychotics, we did a study on 2363 patients of Han Chinese ancestry with schizophrenia.

## Methods

### Study design and participants

Participants included in this study were from the Chinese Antipsychotics Pharmacogenomics Consortium (CAPOC), which aims to investigate the relationship between genetic variants and antipsychotic treatment responses in patients with schizophrenia.

Patients included in this study had a diagnosis of schizophrenia based on the Structured Clinical Interview of DSM-IV, were aged 18–45 years, were of Han Chinese ancestry, scored more than 60 on the Positive and Negative Syndrome Scale (PANSS) (and scored more than four on at least three positive items), were physically healthy with all laboratory parameters within normal limits, had a condition that could be treated with oral medication, and were able to provide informed consent.

Patients were excluded from this study if they had malignant syndrome or acute dystonia, well documented histories of epilepsy and hyperpyretic convulsion, a DSM-IV diagnosis of alcohol or drug dependence, or a history of drug-induced neuroleptic malignant syndrome; required long-acting injectable medication to maintain treatment adherence; were regularly treated with clozapine for treatment resistance during the past month (patients who had taken clozapine for reasons other than treatment resistance were eligible); were treated with electroconvulsive therapy during the last month; had previously attempted suicide, or had experienced the symptoms of severe excitement and agitation; did not have a legal guardian (it was a hospital stipulation that written informed consent was required from the patient’s legal guardian); had QTc prolongation, a history of congenital QTc prolongation, or recent (i.e., within the past 6 months) myocardial infarction; were pregnant or breastfeeding; or had a contraindication to any of the drugs to which they could be assigned.

Participants in this 6-week multicenter randomized open-label pharmacogenetic trial were randomly assigned to seven groups [aripiprazole, olanzapine, quetiapine, risperidone, ziprasidone, or the first-generation antipsychotics (haloperidol or perphenazine)]. All study protocols were approved by the institutional ethics review boards at each site, and written informed consent was obtained. The study was approved by the research ethical committees of local hospitals.

### Clinical assessments and phenotypes

Positive and Negative Syndrome Scale (PANSS) was used to measure symptoms severity of patients with schizophrenia. Assessments were performed by a participating psychiatrist at baseline and at weeks 2, 4, and 6, and their PANSS scores were recorded.

The method of 7 transformed PANSS factors, which has been validated to have minimal between-factor correlations while retaining a high degree of correspondence to standard (Marder) PANSS factors, was applied to evaluate the severity of different symptom domains of schizophrenia, and PANSS scores of participants were transformed into seven factors, i.e., positive, disorganized, negative symptoms apathy/avolition, negative symptoms deficit of expression, hostility, anxiety and depression^10^. To control the effects of baseline severity, percentage changes in PANSS from baseline to week 6 of treatment were calculated to assess treatment responses to antipsychotics^11^. PANSS is an interval scale ranging from 1–7 without a zero point, so we subtracted the theoretical minimum (30 for the total score) from the baseline score when calculating the percentage change in total scores to avoid incorrect calculations^12^. The changes in factors were calculated by using the same method, i.e., subtracting the number of items included in each factor from the factor baseline score. The formulas used to calculate the percentage changes in total scores and factors were as follows.

For total scores,

$${PANSS}\,{per}{centage}\,{change}=\frac{{baseline}\,{total}\,{score}-{week}\,6\,{total}\,{score}}{{\rm{baseline}}\,{\rm{total}}\,{\rm{score}}-30}\times 100$$

for each factor,

$${PANSS}\,{percentage}\,{change}=\frac{{\rm{factor}}\,{\rm{baseline}}\,{\rm{score}}-{\rm{factor}}\,{\rm{week}}\,6\,{\rm{score}}}{{\rm{factor}}\,{\rm{baseline}}\,{\rm{score}}-{\rm{n}}({\rm{items}})}\times 100$$

### Genotyping

**Fig. 1:**
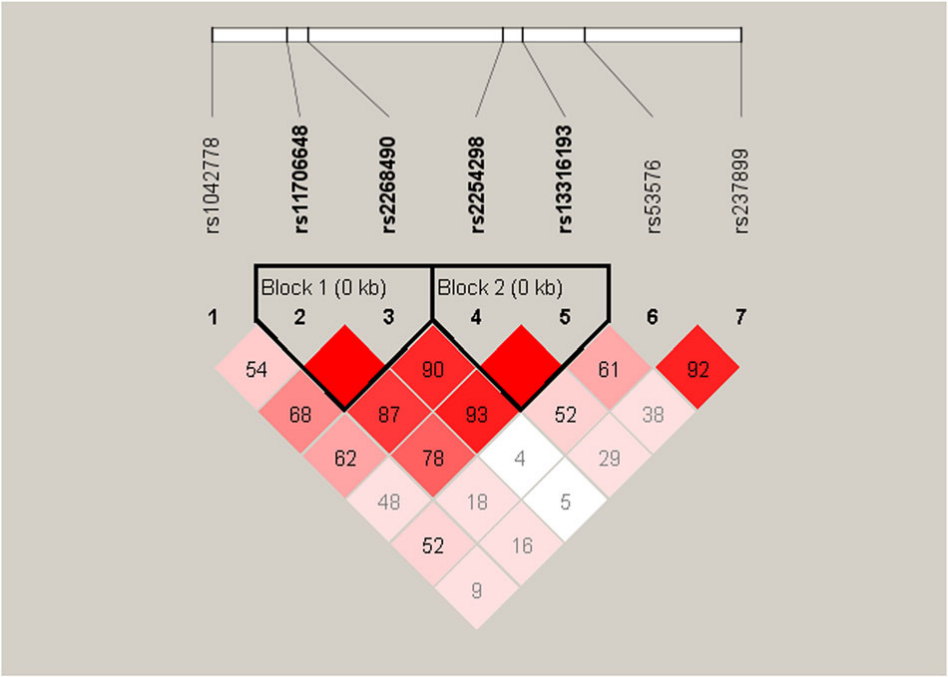
Schematic representation of the 7 selected SNPs on OXTR. The image presented the linkage disequilibrium patterns between seven selected SNPs. Colors indicated the degree of linkage disequilibrium, and numbers indicated the value of 100 multiplied by *D‘*.

As SNPs across the *OXTR* are mostly in high LD with each other, based on previous studies, SNPs (rs53576, rs1042778, rs11706648, rs13316193, rs2254298, rs2268490 and rs237899) were chosen to limit genotyping redundancy (Fig. 1). In total, 5-ml peripheral blood samples from subjects were collected in tubes containing EDTA as an anticoagulant and stored at 4 °C. Genomic DNA was extracted from the samples using a Qiagen QIA amp DNA Mini Kit (Qiagen GmbH, Hilden, Germany) within 1 week and was genotyped with Illumina Human Omni ZhongHua-8 Beadchips (Illumina, San Diego, CA, USA). All genotyping was done blind to the knowledge of the subjects’ clinical data.

### Statistical analysis

All of the statistical analyses were carried out using PLINK 1.9, RStudio 3.6.3 and IBM SPSS Statistics 27 software. Before the analysis, patients’ antipsychotic doses were converted to the equivalent doses of chlorpromazine. Differences in severity and treatment responses of patients with different genotypes were analyzed using two independent-sample *t*-tests or ANOVA appropriately. Associations between the severity of symptoms and *OXTR* polymorphisms and between the treatment responses and *OXTR* polymorphisms were analyzed using the multivariable linear regression, with gender, age, family history, episode status, and the dose of antipsychotics as covariates considering their potential effects. Considering the sexual dimorphism of the oxytocin system, interactions between significantly associated SNPs and gender were examined. Significance was set at *p* < 0.05, and we corrected for multiple testing by using Bonferroni correction.

Given the exploratory nature of this study, for SNPs with an uncorrected *p*-value of less than 0.05 in response association analyses, two-way ANOVA was used to explore the interactions between these SNPs and different antipsychotics; and binary logistic regression was used to explore the associations between these SNPs and response rate with response rate (nonresponse was defined as <50% percentage change).

## Results

### Demographics information of study population

A total of 3030 patients consented to participate in the study, 279 of them did not meet inclusion criteria, 109 of them dropped out before the second week, and 279 of them did not have good-quality DNA available. There were 2363 patients who actually completed 6 weeks of treatment (Table 1), including 1181 males and 1182 females, with an average age of 30.79 ± 7.90 years old. Each SNP and its genetic information for the sample are presented in Table 2. Differences in different genotype groups were investigated within gender, age, family history and medical history, with no statistically significant differences found (Supplementary Table S1). Because of the small sample size of participants with homozygous alternative alleles in rs1042778, rs13316193 and rs237899, homozygous genotype of alternative alleles and heterozygous genotype in these SNPs were combined for analyses and referred to as alternative allele carriers.

**Table 1:** Demographic and clinical characteristics.

	Olanzapine	Risperidone	Quetiapine	Aripiprazole	Ziprasidone	Haploperidol	Perphenazine
	(*N* = 405)	(*N* = 404)	(*N* = 386)	(*N* = 392)	(*N* = 393)	(*N* = 184)	(*N* = 199)
**Sex**							
Male	205 (50.6%)	232 (57.4%)	181 (46.9%)	186 (47.4%)	196 (49.9%)	88 (47.8%)	93 (46.7%)
Female	200 (49.4%)	172 (42.6%)	205 (53.1%)	206 (52.6%)	197 (50.1%)	96 (52.2%)	106 (53.3%)
**Age (years)**							
Mean (SD)	30.1 (±7.89)	30.5 (±7.91)	30.7 (±7.76)	31.0 (±7.82)	30.2 (±7.99)	32.7 (±7.63)	31.9 (±8.04)
**Family history**							
Without	329 (81.2%)	314 (77.7%)	312 (80.8%)	311 (79.3%)	314 (79.9%)	144 (78.3%)	155 (77.9%)
With	76 (18.8%)	90 (22.3%)	74 (19.2%)	81 (20.7%)	79 (20.1%)	40 (21.7%)	44 (22.1%)
**Episode**							
Relapse	291 (71.9%)	278 (68.8%)	283 (73.3%)	278 (70.9%)	284 (72.3%)	138 (75.0%)	147 (73.9%)
First episode	114 (28.1%)	126 (31.2%)	103 (26.7%)	114 (29.1%)	109 (27.7%)	46 (25.0%)	52 (26.1%)
**Duration (months)**							
Mean (SD)	76.1 (±69.0)	74.0 (±69.0)	80.2 (±72.6)	76.3 (±69.2)	72.9 (±68.2)	84.3 (±69.6)	88.9 (±79.0)
**Baseline PANSS score**							
Mean (SD)	88.5 (±15.3)	89.3 (±14.7)	91.0 (±14.9)	89.2 (±14.9)	90.8 (±15.3)	90.4 (±15.8)	92.0 (±15.7)

**Table 2:** Information of selected SNPs.

CHR	SNP	Position	Reference/Alternatives alleles	HWE	MAF	Functional annotation
3	rs1042778	8794545	G > T	0.09373	0.08041	3’ prime UTR variant
3	rs53576	8804371	A > G	0.2717	0.3136	intron variant
3	rs11706648	8796547	A > C	0.6454	0.2791	intron variant
3	rs13316193	8802743	T > C	0.2204	0.1609	intron variant
3	rs2254298	8802228	G > A	0.7292	0.3008	intron variant
3	rs2268490	8797085	C > T	0.2966	0.4628	intron variant
3	rs237899	8808515	G > A	0.2648	0.1149	intron variant

### Association between variants and severity

Analyses of PANSS scores at baseline (Table 3) showed that there were significant differences among genotypes of rs2268490 on scores of positive symptoms (F = 5.364, *p* = 0.005) and scores of negative symptoms apathy/avolition (F = 4.266, *p* = 0.014); total scores (t = −2.479, *p* = 0.013) and scores of hostility symptoms (t = −2.111, *p* = 0.035) were both significantly different between genotypes of rs237899; and scores of anxiety symptoms were significantly different between genotypes of rs13316193 (t = 2.017, *p* = 0.044). While after multiple testing correction, these findings did not reach statistical significance.

**Table 3:** Baseline scores of different schizophrenia symptoms in patients with different genotypes.

		Total		POS		DIS		ANX		HOS		AAA		DOE		DEP	
		Mean	*p (*corrected *p)*	Mean	*p (*corrected *p)*	Mean	*p (*corrected *p)*	Mean	*p (*corrected *p)*	Mean	*p (*corrected *p)*	Mean	*p (*corrected *p)*	Mean	*p (*corrected *p)*	Mean	*p (*corrected *p)*
**rs53576**	AA	89.58	0.067 (1.000)	17.86	0.240 (1.000)	20.37	0.240 (1.000)	4.46	0.517 (1.000)	12.93	0.279 (1.000)	10.08	0.602 (1.000)	11.72	0.456 (1.000)	4.65	0.602 (1.000)
	AG	88.76		17.67		20.17		4.44		12.72		10.01		11.77		4.65	
	GG	91.05		18.03		20.68		4.61		13.15		10.23		12.05		4.77	
**rs11706648**	AA	89.71	0.519 (1.000)	17.75	0.541 (1.000)	20.46	0.287 (1.000)	4.50	0.612 (1.000)	12.91	0.434 (1.000)	10.14	0.258 (1.000)	11.88	0.357 (1.000)	4.68	0.827 (1.000)
	AC	89.08		17.80		20.16		4.42		12.88		9.94		11.65		4.63	
	CC	88.86		18.06		20.20		4.53		12.47		10.23		11.67		4.66	
**rs2254298**	GG	89.18	0.490 (1.000)	17.83	0.857 (1.000)	20.22	0.349 (1.000)	4.47	0.978 (1.000)	12.80	0.525 (1.000)	10.05	0.427 (1.000)	11.77	0.894 (1.000)	4.65	0.717 (1.000)
	GA	89.34		17.78		20.29		4.47		12.96		9.99		11.71		4.65	
	AA	90.49		17.92		20.72		4.50		13.10		10.31		11.82		4.76	
**rs2268490**	CC	89.74	0.068 (1.000)	17.91	0.005* (0.266)	20.29	0.550 (1.000)	4.49	0.416 (1.000)	12.88	0.983 (1.000)	10.16	0.014* (0.794)	11.83	0.775 (1.000)	4.63	0.706 (1.000)
	CT	88.75		17.61		20.19		4.40		12.91		9.88		11.72		4.65	
	TT	90.63		18.26		20.48		4.55		12.92		10.40		11.86		4.73	
**rs1042778**	GG	89.36	0.815 (1.000)	17.79	0.808 (1.000)	20.33	0.702 (1.000)	4.47	0.820 (1.000)	12.84	0.490 (1.000)	10.06	0.848 (1.000)	11.77	0.904 (1.000)	4.66	0.828 (1.000)
	GT/TT	89.55		17.84		20.24		4.49		13.00		10.09		11.79		4.68	
**rs237899**	GG	89.00	0.013* (0.741)	17.77	0.523 (1.000)	20.25	0.166 (1.000)	4.45	0.447 (1.000)	12.76	0.035* (1.000)	10.01	0.164 (1.000)	11.75	0.555 (1.000)	4.64	0.373 (1.000)
	GA/AA	90.76		17.88		20.55		4.53		13.21		10.23		11.86		4.72	
**rs13316193**	TT	89.58	0.243 (1.000)	17.85	0.226 (1.000)	20.41	0.134 (1.000)	4.53	0.044* (1.000)	12.83	0.493 (1.000)	10.05	0.836 (1.000)	11.76	0.982 (1.000)	4.68	0.326 (1.000)
	TC/CC	88.83		17.66		20.10		4.34		12.96		10.02		11.77		4.60	

*POS* positive, *DIS* disorganized, *NAA* negative symptoms apathy/avolition, *NDE* negative symptoms deficit of expression, *HOS* hostility, *ANX* anxiety, *DEP* depression.

*p < 0.05.

The results from the multivariable linear regression analyses (Table 5, Supplementary Table S2) testing the associations among *OXTR* variants and severity of symptoms revealed rs237899 polymorphism was significantly associated with overall symptoms (β = 1.669; *p* = 0.019) and hostility symptoms (β = 0.427; *p* = 0.044); and rs13316193 polymorphism was significantly associated with anxiety symptoms (β = −0.197; *p* = 0.038). These findings did not reach significance after Bonferroni correction. There was no interaction between these SNPs and sex on the severity of symptoms.

### Association between variants and treatment responses

Analyses of percentage changes in PANSS from baseline to week 6 of treatment (Table 4) showed that percentage changes in scores of negative symptoms apathy/avolition were significantly different among genotypes of rs2254298 (F = 3.754, *p* = 0.024). Significant differences were also found among genotypes of rs2268490 on percentage changes in scores of negative symptoms apathy/avolition (F = 3.255, *p* = 0.039). None of the significance, however, remained after multiple testing correction.

**Table 4:** After 6-week treatment with antipsychotics, percentage changes of different schizophrenia symptoms in patients with different genotypes.

		POS		DIS		ANX		HOS		AAA		DOE		DEP	
		Mean	*p (*corrected *p)*	Mean	*p (*corrected *p)*	Mean	*p (*corrected *p)*	Mean	*p (*corrected *p)*	Mean	*p (*corrected *p)*	Mean	*p (*corrected *p)*	Mean	*p (*corrected *p)*
**rs53576**	AA	60.91	0.458 (1.000)	41.01	0.230 (1.000)	65.05	0.841 (1.000)	75.92	0.355 (1.000)	46.83	0.093 (1.000)	45.65	0.366 (1.000)	53.02	0.195 (1.000)
	AG	59.25		39.41		63.74		74.98		43.43		43.46		49.72	
	GG	60.30		41.99		64.37		78.12		45.89		43.35		58.34	
**rs11706648**	AA	59.86	0.603 (1.000)	40.74	0.432 (1.000)	63.69	0.752 (1.000)	75.62	0.917 (1.000)	45.73	0.841 (1.000)	43.66	0.488 (1.000)	51.20	0.270 (1.000)
	AC	60.80		40.53		65.16		76.05		44.82		45.61		51.96	
	CC	58.60		38.02		65.78		75.17		45.28		44.13		60.30	
**rs2254298**	GG	60.33	0.065 (1.000)	39.63	0.278 (1.000)	64.44	0.901 (1.000)	75.78	0.886 (1.000)	44.25	0.024* (1.000)	43.53	0.112 (1.000)	52.96	0.653 (1.000)
	GA	58.78		40.66		64.75		75.40		44.88		44.13		50.31	
	AA	64.16		42.65		62.92		76.53		51.80		49.63		53.39	
**rs2268490**	CC	60.44	0.336 (1.000)	39.44	0.095 (1.000)	66.20	0.581 (1.000)	75.70	0.652 (1.000)	43.85	0.039* (1.000)	44.79	0.146 (1.000)	52.72	0.671 (1.000)
	CT	59.40		40.30		63.47		75.48		44.65		43.85		52.84	
	TT	61.99		42.91		65.27		77.14		49.31		48.13		56.12	
**rs1042778**	GG	59.87	0.291 (1.000)	40.13	0.166 (1.000)	64.42	0.955 (1.000)	75.38	0.166 (1.000)	45.23	0.766 (1.000)	44.51	0.952 (1.000)	51.75	0.495 (1.000)
	GT/TT	61.70		42.20		64.58		77.82		45.84		44.38		54.42	
**rs237899**	GG	60.24	0.790 (1.000)	40.39	0.829 (1.000)	64.77	0.542 (1.000)	75.53	0.481 (1.000)	45.45	0.749 (1.000)	44.55	0.893 (1.000)	51.94	0.756 (1.000)
	GA/AA	59.84		40.67		63.20		76.62		44.87		44.30		53.03	
**rs13316193**	TT	60.18	0.752 (1.000)	40.85	0.153 (1.000)	64.11	0.582 (1.000)	75.59	0.847 (1.000)	45.95	0.120 (1.000)	44.40	0.870 (1.000)	50.67	0.132 (1.000)
	TC/CC	59.75		39.17		65.40		75.86		43.42		44.11		55.55	

*p < 0.05.

The results from the multivariable linear regression analyses (Table 5, Supplementary Table S3) testing the associations among *OXTR* variants and treatment responses revealed rs2268490 polymorphism was significantly associated with negative symptoms apathy/avolition (β = 2.235; *p* = 0.0499). However, this finding did not reach significance after multiple testing correction. There was no interaction between these SNPs and sex on the improvement in symptoms.

**Table 5:** Significant results of analyses on associations with *OXTR* polymorphisms and symptoms of schizophrenia.

	SNP	*β*	95% CI	*P*-value	Corrected *P*-value
**Baseline (score)**					
Total	rs237899	1.669	(0.278, 3.061)	0.019	1.000
Anxiety	rs13316193	−0.197	(−0.383, −0.011)	0.038	1.000
Hostility	rs237899	0.427	(0.012, 0.843)	0.044	1.000
**Treatment response (percentage change)**					
Apathy/Avolition	rs2268490	2.235	(0.0006, 4.469)	0.0499	1.000

**Fig. 2:**
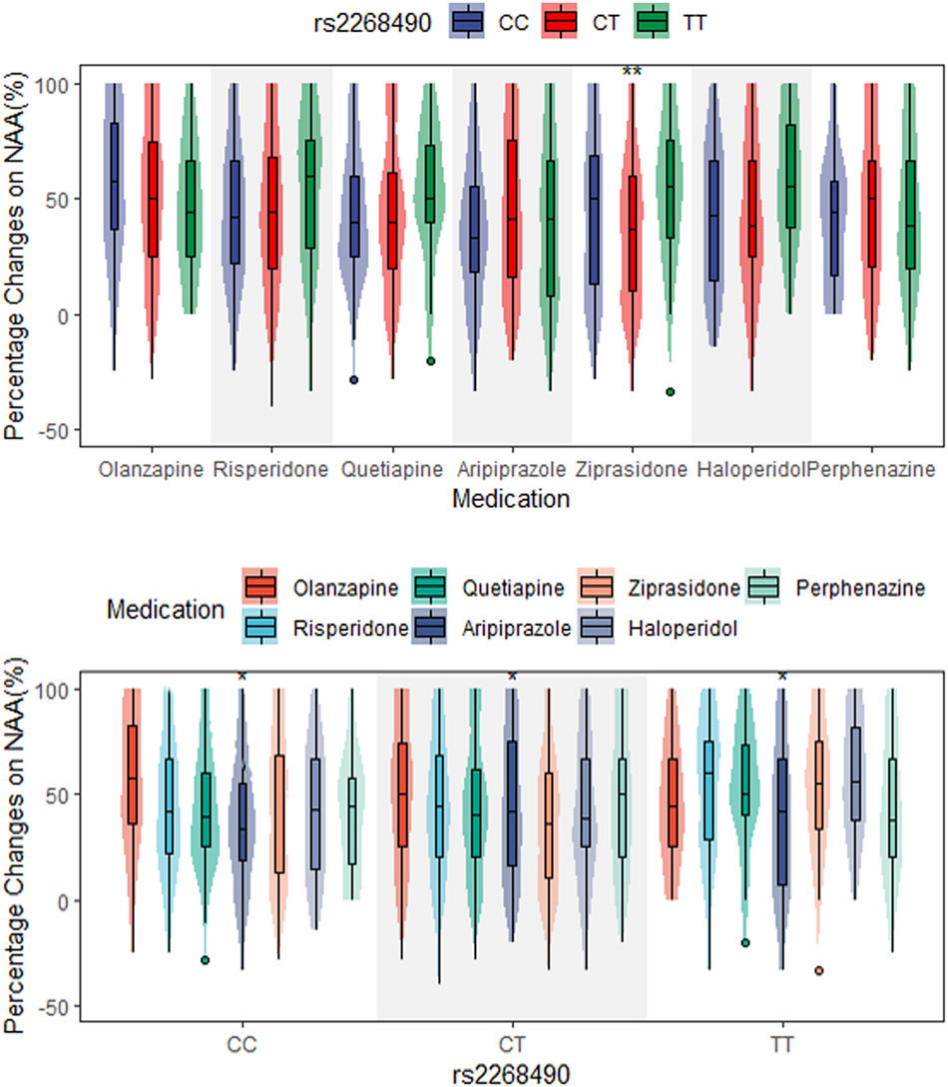
In seven antipsychotic treatment groups, distribution of percentage changes on NAA in patients with different rs2268490 genotypes. *, *p* < 0.05; **, *p* < 0.005.

The results of two-way ANOVA (Table 6, Fig. 2) revealed that there were interactions between rs2268490 and antipsychotics (F = 2.267, *p* = 0.008). Specifically, in the ziprasidone group, patients with rs2268490 TT genotypes had higher percentage changes in negative symptoms apathy/avolition than those with rs2268490 C allele, which reached significance after Bonferroni correction (F = 6.231, *p* = 0.002). Besides, in each genotype group of rs2268490, there were significant differences in patients treated with different antipsychotics on the improvement in negative symptoms apathy/avolition (CC genotype, *p* = 0.017; CT genotype, *p* = 0.034; TT genotype, *p* = 0.038).

**Table 6:** Rs2268490-antipsychotics interaction on the improvement in negative symptoms apathy/avolition.

	Olanzapine	Risperidone	Quetiapine	Aripiprazole	Ziprasidone	Haloperidol	Perphenazine	*F*	*P*
**CC**	55.757	43.034	43.563	36.300	44.395	42.342	40.084	2.586	0.017*
**CT**	51.062	44.672	44.104	44.790	36.861	41.975	48.037	2.277	0.034*
**TT**	45.609	54.325	53.512	39.108	54.646	58.943	44.460	2.229	0.038*
***F***	1.755	2.323	1.810	1.772	6.231	2.318	0.675	2.267	
***P***	0.173	0.098	0.164	0.170	0.002**	0.099	0.509	0.008*	

*p < 0.05; **p < 0.005.

After 6-week treatment, 54.8% of patients with rs2268490 TT genotype got more than 50% improvement in negative symptoms apathy/avolition, while patients with the other rs2268490 genotypes had a lower response rate on this symptom (Supplementary Fig. S1). And rs2268490 showed a significant association with response rate on negative symptoms apathy/avolition (OR = 1.164, *p* = 0.035) (Supplementary Table S4).

### Analyses of eQTL and mQTL

To explore the biological plausibility of *OXTR* in the pathogenesis of schizophrenia, we made eQTL and mQTL analyses using the BRAINEAC and PhenoScanner databases. In the BRAINEAC database (Supplementary Table S5), after Bonferroni correction, rs13316193 was associated with mRNA expression of *OXTR* in the hippocampus (*p* = 5.08 × 10^−^^5^) and temporal cortex (*p* = 3.30 × 10^−^^2^); rs2268490 was associated with mRNA expression of *OXTR* in the hippocampus (*p* = 9.70 × 10^−^^3^) and with mRNA expression of *GRM7* in the frontal cortex (*p* = 3.20 × 10^−^^3^); rs237899 was associated with mRNA expression of *OXTR* in the frontal cortex (*p* = 1.20 × 10^−^^4^), putamen (*p* = 1.70 × 10^−^^2^), temporal cortex (*p* = 1.60 × 10^−^^3^), thalamus (*p* = 1.40 × 10^−^^3^), intralobular white matter (*p* = 5.10 × 10^−^^4^) and across all the tissues (*p* = 2.00 × 10^−^^4^). In the PhenoScanner database (Supplementary Table S6), these three significant SNPs all had mQTL effects in whole blood.

## Discussion

We tested for the associations between *OXTR* polymorphisms and symptoms of schizophrenia. *OXTR* variants were found to be nominally associated with the severity of overall symptoms, hostility symptoms and anxiety symptoms. Regarding treatment responses in schizophrenia, our results showed that *OXTR* rs2268490 presented a nominal association with the improvement in negative symptoms apathy/avolition. Given the exploratory nature of this study, these associations are indicative of the role of the *OXTR* gene in the pathology of schizophrenia though they did not remain significant after multiple testing correction. Besides, the evidence of previous studies and the QTL effects of *OXTR* variants may support plausible explanations of the associations between *OXTR* polymorphisms and schizophrenia.

In our study, *OXTR* rs237899 was found to be associated with the severity of overall symptoms of schizophrenia. Furthermore, the analyses of symptom subdomains of schizophrenia revealed that *OXTR* rs237899 was associated with the severity of hostility symptoms of schizophrenia patients. Specifically, schizophrenia patients with the rs237899 A allele had higher scores on hostility symptoms than those with the rs237899 GG genotype. The hostility symptoms of schizophrenia are characterized by hostility, abnormal excitement, uncooperativeness and poor impulse control. As is shown in our eQTL results, rs237899 has been identified to affect the mRNA expression of OXTR in the frontal cortex, a brain region that was proved to be part of the neuroanatomical model of impulsivity and aggressiveness^13^. Therefore, rs237899 might affect the severity of hostility symptoms through brain functions. Up to now, less research has been done on investigating the association between *OXTR* rs237899 and hostility. Further evidence is needed to clarify the role of *OXTR* rs237899 in hostility.

Anxiety symptoms have been reported to be prevalent in up to 65% of schizophrenia patients^14^ and are related to social functions thereof^15^. Our results suggested that *OXTR* polymorphisms might play a role in the genetic foundation making schizophrenia patients differ in the severity of anxiety symptoms. Specifically, anxiety symptoms of *OXTR* rs13316193 C-carriers were not so severe as those in schizophrenia patients with *OXTR* rs13316193 TT genotype. The mQTL effects of *OXTR* rs13316193 suggested this SNP might affect the methylation of *OXTR* in human blood. As was reported in previous studies, compared to healthy controls, social anxiety disorder patients had lower methylation of *OXTR* in whole blood samples^16^. Thus, *OXTR* polymorphisms may influence the susceptibility to anxiety in patients with different genotypes through methylation of *OXTR*.

Compared to positive symptoms, apathy/avolition symptoms in schizophrenia patients tend to be associated with a poorer prognosis and are related to more genetic vulnerability for schizophrenia^17,18^. We found that *OXTR* rs2268490 was associated with schizophrenia patients’ treatment responses to antipsychotics. Specifically, there were significant differences in the improvement in negative symptoms apathy/avolition among genotypes of rs2268490 in patients treated with ziprasidone. Previous studies indicated the associations between *OXTR* rs2268490 and prosocial behaviors^19–21^; however, studies about the associations between this SNP and the improvement in negative symptoms apathy/avolition are lacking. Research on the pathology of apathy/avolition symptoms has proposed that deficits of the hedonic pathway provide foundations for these symptoms and that glutamate is one of the crucial molecules involved in the hedonic pathway^22^. As is shown in our results, in the frontal cortex, there are eQTL associations of rs2268490 with metabotropic glutamate receptor 7 (*GRM7*), a critical actor in regulating synaptic glutamate transmission^23^. Besides, *GRM7* has been found to be associated with the risk of schizophrenia and the treatment responses of antipsychotics^24^. Therefore, these findings may explain the biological mechanism of the differences in responses to antipsychotics in patients with different rs2268490 genotypes.

At present, the main treatment for schizophrenia is antipsychotics, which work mainly by blocking the D2 family of postsynaptic dopamine receptors. It is notable that the oxytocin system has been found to interact with the dopamine system. Dopamine d2-oxytocin receptor heteromers in the ventral and dorsal striatum are essential molecules involved in oxytocin-induced changes in social and behaviors^25^. Moreover, studies on hypothalamic oxytocinergic projections support evidence that there are interactions between oxytocin systems and dopamine circuits^26^. Therefore, *OXTR* polymorphisms may influence the severity and treatment responses of schizophrenia symptoms by interactions with the dopamine system.

To sum up, our results highlighted the possibility of nominally significant associations of *OXTR* gene polymorphisms with the severity and improvement in schizophrenia symptoms. Several factors may be contributing to the results that no SNP survived multiple correction for association. First, based on the hypothesis that susceptibility to schizophrenia is determined by variation in many genes of small effect, each individual variant was likely to have been underpowered to observe an effect. Therefore, further research is needed to explore the interaction in candidate genes of schizophrenia. Second, most previous studies were conducted in the European population and it can be expected that some of the findings about the associations between the *OXTR* gene and schizophrenia may be population-specific. More large-scale datasets collected from global populations may help to understand more about the population-specific heterogeneity of *OXTR* genetic effects. Third, potential confounding factors were not completely ruled out. Future studies could incorporate more covariates, such as medication history, allowing for a more comprehensive analysis of the relationships between the *OXTR* gene and schizophrenia.

To our knowledge, this is the largest study to investigate how *OXTR* polymorphisms impact the symptoms and treatment responses of schizophrenia. Besides, the kinds of antipsychotics used in our study went beyond related published research. Third, we tested the effects of *OXTR* polymorphisms on different dimensions, making it possible to further understand the mechanisms of different symptoms. There were several limitations in our study. Although considering underlying non-genetic factors and using them as covariates, other potential environmental factors like family support and socioeconomic status, which have been reported to play joint roles with *OXTR* in regulating the physical and mental state of humans^27,28^, should be analyzed in further studies. In addition, the sample size of each antipsychotic is relatively small, therefore, the interactions between rs2268490 and antipsychotics on the improvement in negative symptoms apathy/avolition warrant replication.

In summary, we have identified that *OXTR* polymorphisms were nominally associated with the severity of overall symptoms, hostility symptoms and anxiety symptoms of schizophrenia and with the improvement in negative symptoms apathy/avolition and hostility symptoms. Besides, we have found that patients with different rs2268490 genotypes significantly differed in the improvement in negative symptoms apathy/avolition when treated with ziprasidone. These findings may contribute to further elucidate the mechanism of specific symptoms of schizophrenia and to exploit antipsychotics more effective to specific symptoms.

### Supplementary information

Supplementary materials

## Data Availability

The data supporting the findings of this study are available from the corresponding author upon reasonable request. Requests to access these datasets should be directed to W.-H.Y., dryue@bjmu.edu.cn.
