# Author Correction: *OXTR* polymorphisms associated with severity and treatment responses of schizophrenia

**DOI:** 10.1038/s41537-024-00436-6

**Published:** 2024-02-03

**Authors:** Xue Lv, Yue-Sen Hou, Zhao-Hui Zhang, Wei-Hua Yue

**Affiliations:** 1 The First Affiliated Hospital of Xinxiang Medical College, 453100 Xinxiang, Henan, China; 2 Henan Key Laboratory of Neurorestoratology, 453199 Xinxiang, Henan China; 3 Henan Engineering Research Center of Physical Diagnostics and Treatment Technology for the Mental and Neurological Diseases, 453005 Xinxiang, Henan China; 4 Peking University Sixth Hospital, Peking University Institute of Mental Health, 100191 Beijing, China

**Keywords:** Genetics of the nervous system, Schizophrenia

Correction to: *Schizophrenia* 10.1038/s41537-023-00413-5, published online 06 January 2024

“In the Acknowledgements section, ‘This work was supported by grants from the Postgraduate Education Reform and’ Quality Improvement Project of Henan Province (YJS2021JD12) and YJSCX202275Y’ should have read ‘This work was supported by grants from the National Natural Science Foundation of China (81825009); National Key R&D Program of China (2021YFF1201103); CAMS Innovation Fund for Medical Sciences (2021-I2M-C&T-B-099, 2019-I2M-5-006); Chinese Institute for Brain Research at Beijing (2020-NKX-XM-12); PKUHSC-KCL Joint Medical Research (BMU2020KCL001); National Natural Science Foundation of China (81901358); the Postgraduate Education Reform and Quality Improvement Project of Henan Province (YJS2021JD12) and YJSCX202275Y’. The original article has been corrected.”

