# Supplementary material for: OXTR polymorphisms associated with severity and treatment responses of schizophrenia
Source: Schizophrenia (Heidelb). 2024 Jan 6;10(1):7. doi: 10.1038/s41537-023-00413-5 (PMC10851696; doi:10.1038/s41537-023-00413-5)
Supplement: Supplementary file 1 — Supplementary materials [file 41537_2023_413_MOESM1_ESM.docx]

**Supplementary materials**

**Table S1** Demographic and clinical characteristics in patients with different genotypes.

|  |  |  | **Sex** | |  | **Age (years)** |  | **Family history** | |  | **Episode** | |  | **Duration (months)** |
| --- | --- | --- | --- | --- | --- | --- | --- | --- | --- | --- | --- | --- | --- | --- |
|  |  |  | Male | Female |  |  |  | Without | With |  | Relapse | First episode |  |  |
| **rs53576** | AA (N=1125) |  | 577 (51.3 %) | 548 (48.7 %) |  | 30.9 (± 7.91) |  | 878 (78.0 %) | 247 (22.0 %) |  | 812 (72.2 %) | 313 (27.8 %) |  | 77.7 (± 71.2) |
|  | AG (N=994) |  | 487 (49.0 %) | 507 (51.0 %) |  | 30.6 (± 7.82) |  | 807 (81.2 %) | 187 (18.8 %) |  | 712 (71.6 %) | 282 (28.4 %) |  | 78.1 (± 69.7) |
|  | GG (N=244) |  | 117 (48.0 %) | 127 (52.0 %) |  | 31.0 (± 8.12) |  | 194 (79.5 %) | 50 (20.5 %) |  | 175 (71.7 %) | 69 (28.3 %) |  | 75.3 (± 70.8) |
| **rs11706648** | AA (N=1223) |  | 623 (50.9 %) | 600 (49.1 %) |  | 30.7 (± 7.88) |  | 969 (79.2 %) | 254 (20.8 %) |  | 876 (71.6 %) | 347 (28.4 %) |  | 76.1 (± 68.8) |
|  | AC (N=961) |  | 474 (49.3 %) | 487 (50.7 %) |  | 30.9 (± 7.95) |  | 770 (80.1 %) | 191 (19.9 %) |  | 700 (72.8 %) | 261 (27.2 %) |  | 79.5 (± 73.0) |
|  | CC (N=179) |  | 84 (46.9 %) | 95 (53.1 %) |  | 31.0 (± 7.72) |  | 140 (78.2 %) | 39 (21.8 %) |  | 123 (68.7 %) | 56 (31.3 %) |  | 77.4 (± 68.5) |
| **rs2254298** | GG (N=1125) | | 568 (50.5 %) | 557 (49.5 %) |  | 30.9 (± 7.90) |  | 898 (79.8 %) | 227 (20.2 %) |  | 815 (72.4 %) | 310 (27.6 %) |  | 79.6 (± 71.5) |
|  | GA (N=979) |  | 472 (48.2 %) | 507 (51.8 %) |  | 30.9 (± 7.95) |  | 776 (79.3 %) | 203 (20.7 %) |  | 703 (71.8 %) | 276 (28.2 %) |  | 78.6 (± 71.2) |
|  | AA (N=205) |  | 112 (54.6 %) | 93 (45.4 %) |  | 29.9 (± 7.75) |  | 167 (81.5 %) | 38 (18.5 %) |  | 142 (69.3 %) | 63 (30.7 %) |  | 63.6 (± 59.2) |
| **rs2268490** | CC (N=578) |  | 261 (45.2 %) | 317 (54.8 %) |  | 31.5 (± 7.84) |  | 464 (80.3 %) | 114 (19.7 %) |  | 419 (72.5 %) | 159 (27.5 %) |  | 81.3 (± 70.4) |
|  | CT (N=953) |  | 501 (52.6 %) | 452 (47.4 %) |  | 30.8 (± 7.95) |  | 753 (79.0 %) | 200 (21.0 %) |  | 690 (72.4 %) | 263 (27.6 %) |  | 79.0 (± 71.7) |
|  | TT (N=432) |  | 219 (50.7 %) | 213 (49.3 %) |  | 30.6 (± 7.94) |  | 344 (79.6 %) | 88 (20.4 %) |  | 310 (71.8 %) | 122 (28.2 %) |  | 73.9 (± 67.8) |
| **rs1042778** | GG (N=1992) | | 993 (49.8 %) | 999 (50.2 %) |  | 30.8 (± 7.92) |  | 1585 (79.6 %) | 407 (20.4 %) |  | 1436 (72.1 %) | 556 (27.9 %) |  | 77.6 (± 70.4) |
|  | GT (N=362) |  | 183 (50.6 %) | 179 (49.4 %) |  | 30.6 (± 7.76) |  | 287 (79.3 %) | 75 (20.7 %) |  | 255 (70.4 %) | 107 (29.6 %) |  | 77.8 (± 71.4) |
|  | TT (N=9) |  | 5 (55.6 %) | 4 (44.4 %) |  | 31.0 (± 8.12) |  | 7 (77.8 %) | 2 (22.2 %) |  | 8 (88.9 %) | 1 (11.1 %) |  | 64.3 (± 48.8) |
| **rs237899** | GG (N=1845) | | 899 (48.7 %) | 946 (51.3 %) |  | 30.8 (± 7.89) |  | 1460 (79.1 %) | 385 (20.9 %) |  | 1314 (71.2 %) | 531 (28.8 %) |  | 77.8 (± 70.9) |
|  | GA (N=493) |  | 267 (54.2 %) | 226 (45.8 %) |  | 30.7 (± 7.93) |  | 400 (81.1 %) | 93 (18.9 %) |  | 364 (73.8 %) | 129 (26.2 %) |  | 76.8 (± 69.1) |
|  | AA (N=25) |  | 15 (60.0 %) | 10 (40.0 %) |  | 29.8 (± 8.05) |  | 19 (76.0 %) | 6 (24.0 %) |  | 21 (84.0 %) | 4 (16.0 %) |  | 80.0 (± 72.4) |
| **rs13316193** | TT (N=1641) |  | 824 (50.2 %) | 817 (49.8 %) |  | 30.8 (± 7.87) |  | 1301 (79.3 %) | 340 (20.7 %) |  | 1170 (71.3 %) | 471 (28.7 %) |  | 78.1 (± 71.4) |
|  | TC (N=650) |  | 317 (48.8 %) | 333 (51.2 %) |  | 30.9 (± 7.99) |  | 523 (80.5 %) | 127 (19.5 %) |  | 477 (73.4 %) | 173 (26.6 %) |  | 77.0 (± 68.5) |
|  | CC (N=52) |  | 29 (55.8 %) | 23 (44.2 %) |  | 30.1 (± 7.52) |  | 42 (80.8 %) | 10 (19.2 %) |  | 37 (71.2 %) | 15 (28.8 %) |  | 80.1 (± 66.0) |

**Table S2** Results of associations analyses between *OXTR* polymorphisms and the severity of schizophrenia symptoms.

|  | **Total** | | **POS** | | **DIS** | | **ANX** | | **HOS** | | **NAA** | | **DOE** | | **DEP** | |
| --- | --- | --- | --- | --- | --- | --- | --- | --- | --- | --- | --- | --- | --- | --- | --- | --- |
|  | *p*-value | Corrected *P*-value | *p*-value | Corrected *P*-value | *p*-value | Corrected *P*-value | *p*-value | Corrected *P*-value | *p*-value | Corrected *P*-value | *p*-value | Corrected *P*-value | *p*-value | Corrected *P*-value | *p*-value | Corrected *P*-value |
| **rs1042778** | 0.791 | 1.000 | 0.765 | 1.000 | 0.718 | 1.000 | 0.816 | 1.000 | 0.464 | 1.000 | 0.874 | 1.000 | 0.917 | 1.000 | 0.854 | 1.000 |
| **rs237899** | 0.019 | 1.000 | 0.424 | 1.000 | 0.195 | 1.000 | 0.409 | 1.000 | 0.044 | 1.000 | 0.218 | 1.000 | 0.622 | 1.000 | 0.318 | 1.000 |
| **rs13316193** | 0.236 | 1.000 | 0.188 | 1.000 | 0.133 | 1.000 | 0.038 | 1.000 | 0.539 | 1.000 | 0.883 | 1.000 | 0.914 | 1.000 | 0.323 | 1.000 |
| **rs53576** | 0.636 | 1.000 | 0.782 | 1.000 | 0.802 | 1.000 | 0.634 | 1.000 | 0.963 | 1.000 | 0.686 | 1.000 | 0.218 | 1.000 | 0.523 | 1.000 |
| **rs11706648** | 0.256 | 1.000 | 0.384 | 1.000 | 0.139 | 1.000 | 0.659 | 1.000 | 0.346 | 1.000 | 0.518 | 1.000 | 0.184 | 1.000 | 0.639 | 1.000 |
| **rs2254298** | 0.239 | 1.000 | 0.933 | 1.000 | 0.136 | 1.000 | 0.951 | 1.000 | 0.248 | 1.000 | 0.391 | 1.000 | 0.842 | 1.000 | 0.648 | 1.000 |
| **rs2268490** | 0.410 | 1.000 | 0.140 | 1.000 | 0.510 | 1.000 | 0.799 | 1.000 | 0.828 | 1.000 | 0.362 | 1.000 | 0.878 | 1.000 | 0.463 | 1.000 |

**Table S3** Results of associations analyses between *OXTR* polymorphisms and the treatment responses of schizophrenia symptoms.

|  | **Total** | | **POS** | | **DIS** | | **ANX** | | **HOS** | | **NAA** | | **DOE** | | **DEP** | |
| --- | --- | --- | --- | --- | --- | --- | --- | --- | --- | --- | --- | --- | --- | --- | --- | --- |
|  | *p*-value | Corrected *P*-value | *p*-value | Corrected *P*-value | *p*-value | Corrected *P*-value | *p*-value | Corrected *P*-value | *p*-value | Corrected *P*-value | *p*-value | Corrected *P*-value | *p*-value | Corrected *P*-value | *p*-value | Corrected *P*-value |
| **rs1042778** | 0.304 | 1.000 | 0.349 | 1.000 | 0.193 | 1.000 | 0.969 | 1.000 | 0.208 | 1.000 | 0.744 | 1.000 | 0.885 | 1.000 | 0.517 | 1.000 |
| **rs237899** | 0.928 | 1.000 | 0.741 | 1.000 | 0.819 | 1.000 | 0.579 | 1.000 | 0.429 | 1.000 | 0.798 | 1.000 | 0.866 | 1.000 | 0.812 | 1.000 |
| **rs13316193** | 0.400 | 1.000 | 0.587 | 1.000 | 0.088 | 1.000 | 0.626 | 1.000 | 0.916 | 1.000 | 0.126 | 1.000 | 0.732 | 1.000 | 0.174 | 1.000 |
| **rs53576** | 0.196 | 1.000 | 0.434 | 1.000 | 0.671 | 1.000 | 0.676 | 1.000 | 0.628 | 1.000 | 0.162 | 1.000 | 0.153 | 1.000 | 0.800 | 1.000 |
| **rs11706648** | 0.912 | 1.000 | 0.771 | 1.000 | 0.408 | 1.000 | 0.444 | 1.000 | 0.865 | 1.000 | 0.908 | 1.000 | 0.304 | 1.000 | 0.196 | 1.000 |
| **rs2254298** | 0.383 | 1.000 | 0.896 | 1.000 | 0.229 | 1.000 | 0.839 | 1.000 | 0.928 | 1.000 | 0.095 | 1.000 | 0.180 | 1.000 | 0.681 | 1.000 |
| **rs2268490** | 0.210 | 1.000 | 0.682 | 1.000 | 0.062 | 1.000 | 0.740 | 1.000 | 0.502 | 1.000 | 0.0499 | 1.000 | 0.298 | 1.000 | 0.414 | 1.000 |

**Table S4** Result of binary logistic regression exploring the associations between *OXTR* rs2268490 and response rate of negative symptoms apathy/avolition.

|  | **SNP** | **OR** | **95% CI** | ***P*-value** |
| --- | --- | --- | --- | --- |
| NAA | rs2268490 | 1.164 | (1.011, 1.341) | 0.035 |

**Table S5** The eQTL analysis results for significant SNPs from the published human brain regions cis-eQTL database.

| **Gene Symbol** | **marker** | **rsid** | **Expr ID** | **ave ALL** | **CRBL** | **FCTX** | **HIPP** | **MEDU** | **OCTX** | **PUTM** | **SNIG** | **TCTX** | **THAL** | **WHMT** |
| --- | --- | --- | --- | --- | --- | --- | --- | --- | --- | --- | --- | --- | --- | --- |
| OXTR | chr3:8802743 | rs13316193 | 2662008 | 6.70E-01 | 2.80E-01 | 5.20E-02 | 4.50E-02 | 6.50E-01 | 1.00E+00 | 8.70E-01 | 9.30E-01 | 3.30E-02 | 3.50E-01 | 1.10E-01 |
| OXTR | chr3:8797085 | rs2268490 | 2662008 | 3.30E-01 | 8.60E-01 | 2.80E-01 | 9.70E-03 | 6.50E-01 | 6.40E-01 | 8.90E-01 | 5.70E-01 | 9.90E-02 | 1.40E-01 | 2.50E-01 |
| GRM7 | chr3:8797085 | rs2268490 | 2609174 | 2.70E-01 | 9.10E-01 | 3.20E-03 | 4.50E-01 | 7.90E-01 | 8.20E-01 | 7.70E-01 | 7.70E-01 | 5.60E-01 | 4.90E-01 | 7.00E-01 |
| OXTR | chr3:8808515 | rs237899 | t2661992 | 2.00E-04 | 1.20E-01 | 1.20E-04 | 1.80E-01 | 1.40E-01 | 8.50E-02 | 1.70E-02 | 5.50E-01 | 1.60E-03 | 1.40E-03 | 5.10E-04 |

**Table S6** The mQTL analysis results for significant SNPs from PhenoScanner database.

| **rsid** | **trait** | **efo** | **study** | **pmid** | **tissue** | **marker** | **location** | ***p*-vlaue** |
| --- | --- | --- | --- | --- | --- | --- | --- | --- |
| rs2268490 | DNA methylation | GO_0006306 | BIOSQTL | 27918535 | Whole blood | cg16448890 | chr3:8783589 | 5.05E-23 |
| rs237899 | DNA methylation | GO_0006306 | BIOSQTL | 27918535 | Whole blood | cg00385883 | chr3:8808235 | 9.26E-94 |
| rs13316193 | DNA methylation | GO_0006306 | BIOSQTL | 27918535 | Whole blood | cg00078085 | chr3:8810616 | 5.12E-20 |


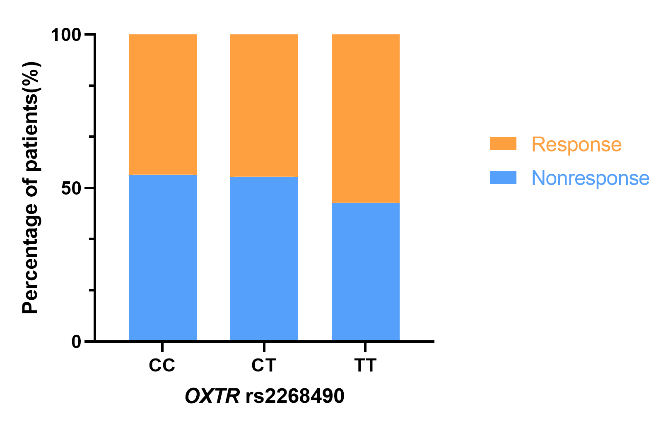


**Figure S1.** Response rate of patients with *OXTR* rs2268490 genotypes on negative symptoms apathy/avolition.
